# Tocilizumab in the treatment of coronavirus disease 2019 pneumonia: real-world data from a case series

**DOI:** 10.2217/fvl-2020-0410

**Published:** 2021-05-24

**Authors:** Ran Cui, Ying Zhu, Yulan Wang, Xiao-Hua Chen, Qiang Li, Sheng-Ming Dai, Qiang Tong

**Affiliations:** 1 ^1^Department of Rheumatology & Immunology, Shanghai Jiao Tong University Affiliated Sixth People’s Hospital, Shanghai, China; 2 ^2^Department of Infectious Diseases, Wuhan Leishenshan Hospital, Wuhan, Hubei, China; 3 ^3^Department of Respiratory & Critical Care Medicine, Seventh Medical Center of Chinese PLA General Hospital, Beijing, China; 4 ^4^Molecular Diagnostic Laboratory of Cancer Center, Shanghai General Hospital, Shanghai Jiao Tong University School of Medicine, Shanghai, China; 5 ^5^Department of Infectious Diseases, Shanghai Jiao Tong University Affiliated Sixth People’s Hospital, Shanghai, China; 6 ^6^Department of Respiratory & Critical Care Medicine, Shanghai East Hospital, Tongji University School of Medical, Shanghai, China

**Keywords:** adverse events, COVID-19, cytokine release syndrome, IL-6, IL-6R antagonists, IL-6 receptor, inflammatory response, mortality, SARS-CoV-2, tocilizumab

## Abstract

**Aim:** Coronavirus disease 2019 is a life-threatening disease and how to improve survival of the patients is of great importance. **Objective:** To determine whether tocilizumab (TCZ) shows favorable results in coronavirus disease 2019 patients. **Materials & methods:** A retrospective study of four patients who received TCZ was conducted from 19 February to 31 March 2020 at Leishenshan Hospital, Wuhan, China. Clinical data of patients were compared before and after the administration of the agent. **Results:** There was not much difference in the clinical feature improvements and computed tomography images after TCZ administration in two mild patients. The other two severe patients died of disseminated intravascular coagulation and acute respiratory distress syndrome, respectively. **Conclusion:** Administration of TCZ was not shown a favorable outcome in this preliminary uncontrolled case series.

## Lay abstract

Patients treated with tocilizumab was not indicated a favoring outcome with not much improvement in two moderate patients and two severe patients died. Early identification of cytokine release syndrome in treating coronavirus disease 2019 patients with IL-6 antagonist is required.

A pandemic of coronavirus disease 2019 (COVID-19) caused by SARS-CoV-2 has swept over worldwide. It is reported that approximately 17–29% of infected people with chronic comorbidities may rapidly deteriorate into acute respiratory distress syndrome (ARDS) and septic shock, 11–15% of which die of multiple organ failure [1,2]. So far, no specific therapeutic agent has been reported to decrease mortality substantially. A clinical trial of lopinavir–ritonavir in adults with severe novel coronavirus pneumonia (NCP) was found to have no beneficial outcomes [3]. Other potential therapies, such as remdesivir, hydroxychloroquine, convalescent plasma, etc., are still under investigation [4–6]. Tocilizumab (TCZ), an inhibitor of IL-6 receptor, was approved for the treatment of cytokine-release syndrome (CRS) by the US FDA, and it has been recommended as an optional therapy in the treatment of severe patients with COVID-19 in the seventh version of the guidelines published by the National Health Commission of China [7]. A small study of 21 patients concluded that TCZ is effective in treating severe cases, although there is still a paucity of data assessing the efficacy and safety of TCZ [8]. Later on, more publications reported that patients can benefit partially or totally from TCZ treatment [9–11]. However, in the very beginning when we applied TCZ treatment to the COVID-19 patients, we found some opposite findings. We herein report the cases series of four patients with COVID-19 in the administration of TCZ in the real world.

## Materials & methods

The study was conducted at the Infectious Disease Department, Leishenshan Hospital, Wuhan, China, from 19 February to 31 March 2020, and the final follow-up date was 31 March 2020. The study was approved by the ethics committees as required from the hospital. Written informed consent was obtained from each patient involved or their family member.

## Patients

Patients confirmed to have COVID-19 by a positive PCR test when they were first diagnosed were eligible to receive TCZ if they fulfilled the following criteria: they were severe patients for whom computed tomography (CT) images showed bilateral diffuse lung disease, their serum level of IL-6 was detected as being elevated continuously high.

## Disease severity classification

Two patients were classified as the moderate type. This type experiences the following conditions: fever; respiratory symptoms; pneumonia performance on x-rays or CT scans.

Two patients were considered as the severe type in admission and rapidly progressed to the critical type. Patients are classified as the severe type if they meet any of the following criteria: respiratory distress (RR ≥30/min); oxygen saturation ≤93% when breathing ambient air; arterial partial pressure of oxygen (PaO_2_)/fraction of inspired oxygen (FiO_2_) ≤300 mmHg.

Patients are classified as the critical type if they satisfy any of the following conditions: respiratory failure requiring mechanical ventilation; shock; failure of other organs requiring intensive care unit monitoring and management.

## Clinical information

Clinical features, radiological abnormalities, and laboratory results were collected before and after TCZ treatment. Clinical features included: demographic data, basic information, days of admission from symptom onset and presenting symptoms, data related to the therapy plan and changes in clinical manifestations. Radiological abnormalities were assessed by x-ray and chest CT image. Laboratory data included white blood cell count, lymphocyte count, chemistry panels with liver and kidney function, inflammatory factors C-reactive protein (CRP), erythrocyte sedimentation rate and cytokines IL-6, TNF-α and IL-10.

## Results

In accordance with the National Health Commission of China’s guidelines, patients were admitted from 19 February 2020 to 29 February 2020. Two moderate, one severe and one critical patient met the diagnosis and treatment protocol for NCP defined in the seventh edition of the guidelines (demographic characteristics shown in Table 1). The initial symptoms were fever (4/4 patients), cough (3/4), sputum production (1/4), chest tightness (1/4), tachypnea (1/4), shortness of breath (4/4), fatigue (3/4), myalgia (1/4) and poor appetite (1/4). Symptoms of Cases 1 and 2 were mostly resolved, except for cough and remittent fever after TCZ management. Not much amelioration of symptoms was seen in Cases 3 and 4. As for comorbidities, diabetes was shown in three patients and hypertension in one. Moderate patients (Case 1 and Case 2) had diabetes and emphysema, respectively. The severe patient (Case 3) had a history of chronic hepatitis and diffuse large B-cell lymphoma. He had a long-term medication of entecavir and had undergone chemotherapy in combination with rituximab. Case 4 was a critically ill patient who had a history of hypertension and diabetes with an unstable condition.

**Table 1.** Demographic characteristics of patients with novel coronavirus pneumonia.

	Moderate cases in non-ICU		Severe and critically severe cases in ICU	
	Case 1	Case 2	Case 3	Case 4
Age	69	64	47	73
Gender	Female	Male	Male	Male
Date of onset of symptoms	6 January 2020	18 January 2020	12 February 2020	4 February 2020
Date of admission	29 February 2020	29 February 2020	29 February 2020	19 February 2020
Duration time (day)	55	43	18	16
Initial symptoms				√
Fever (°C)	37.8	40	40	39
Cough	√	√	√	
Sputum production				
Chest tightness			√	
Tachypnea			√	
Shortness of breath	√	√	√	√
Fatigue		√	√	√
Myalgia				√
Poor appetite			√	
Comorbidities				
Hypertension				√
Diabetes	√		√	√
Emphysema		√		
Hepatitis			√	
DLBCL			√	

DLBCL: Diffuse large B-cell lymphoma; ICU: Intensive care unit; NCP: Novel coronavirus pneumonia.

Nasopharyngeal or oropharyngeal swab specimens were collected in all four cases, and the results were negative on admission, while positive when they were first diagnosed.

Because of an inadequate response to standard treatments in two moderate patients (Cases 1 and 2), TCZ (Roche Pharma [Schweiz] Ltd, Basel, Switzerland) was administered in 400 mg doses intravenously once for each patient (illness day 59 or hospital day 5 of case 1 and illness day 47 or hospital day 5 of case 2). The severe and critically ill patients (Cases 3 and 4) with high levels of IL-6 were administered TCZ at a dose of 8 mg/kg (672 mg of Case 3 in day 33 of symptom onset or hospital day 8 and 600 mg once of Case 4 in day 23 of symptom onset or hospital day 18) after failing to respond to the treatments of antiviral, antibiotics, immunomodulator and corticosteroid (See details in Table 2.) All patients were given oxygen supported by nasal cannula or BiPAP mask. Mechanical ventilation was used in two severe patients, and ECMO was applied to the Case 3 patient, whose condition developed rapidly to critical during the course of the disease.

**Table 2.** Treatment and outcome of patients with novel coronavirus pneumonia.

	Moderate cases in non-ICU		Critically severe cases in ICU	
	Case 1	Case 2	Case 3	Case 4
Treatment				
Antiviral	Arbidol hydrochloride + Lianhua Qingwen Capsule	Arbidol hydrochloride + Lianhua Qingwen Capsule	Arbidol hydrochloride	Lianhua Qingwen Capsule + ribavirin (0.5 g qd)
Antibiotics		Moxifoxacin (0.4 qd)	Meropenem (2 g q8h) + vancomycin (0.5 g q12h)	Imipenem (1 g q12h) + vancomycin (0.5 g q12h) + tigercyclin (vancomycin discontinued, 100 mg q12h) + caspofungin (50 mg, qd) + voriconazole (caspofungin discontinued, 0.2 g q12h)
Immunomodulator		Thymalfasin (1.6 mg biw)		Thymalfasin (1.6 mg qw)
Corticosteroid		Methylprednisolone (40 mg qd)	Methylprednisolone (40 mg qd)	Methylprednisolone (40 mg bid)
Nutrition support	Human albumin (10 g once)	Human albumin (10 g qd)	Human albumin (30 g qd) + human immunoglobulin (10 g once)	Human albumin (10 g qd) + human immunoglobulin (10 g once)
Tocilizumab	Tocilizumab (400 mg once) (illness day 59 or hospital day 5)	Tocilizumab (400 mg once) (illness day 47 or hospital day 5)	Tocilizumab (400 + 272 mg) (illness day 33 or hospital day 8)	Tocilizumab (600 mg once) (illness day 23 or hospital day 18)
Oxygen support	Nasal cannula (2 l/min)	Nasal cannula (2 l/min)	Nasal cannula 2 l/min + BiPAP mask	
Mechanical ventilation				Invasive mechanical ventilation
ECMO			ECMO	
Other supportive treatment			Suspended RBC (2 U)	Closed thoracic drainage
CT image	Progressed	Progressed	Progressed	Progressed
Outcome	Stable	Stable	Die of DIC	Die of ARDS

ARDS: Acute respiratory distress syndrome; CT: Computed tomography; DIC: Disseminated intravascular coagulation; ECMO: Extracorporeal membrane oxygenation; ICU: Intensive care unit; NCP: Novel coronavirus pneumonia; qd: Once a day; qw: Once a week; RBC: Red blood cells.

**Figure 1.**
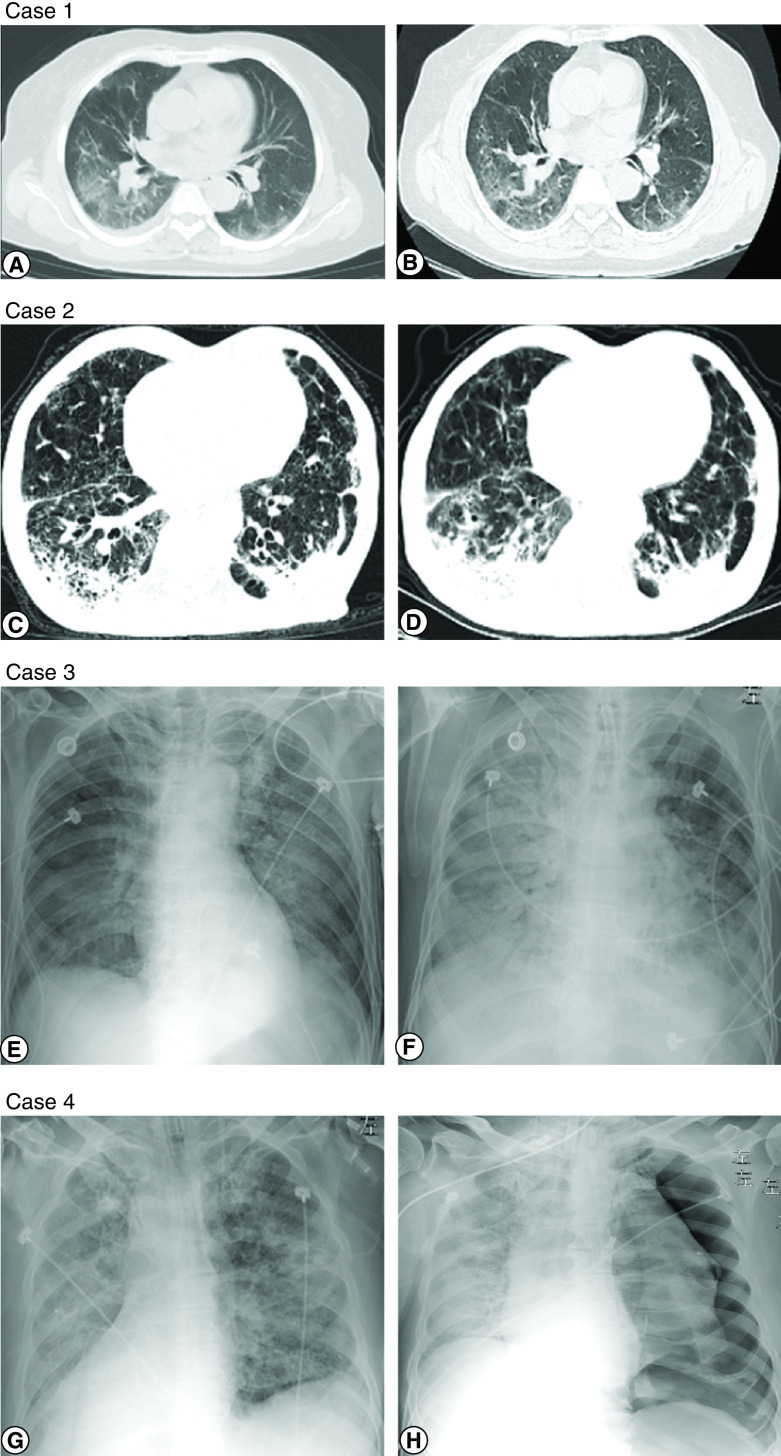
Radiological images of four patients before and after tocilizumab administration. Case 1, a 69-year-old woman with diabetes **(A)** 59 days after symptom onset: multiple peripheral patchy ground-glass opacity with a small pleural effusion in the right lung **(B)** after tocilizumab administration (illness day 67): bilateral peripheral streaky opacities with internal bronchovascular bundle thickening. Case 2, a 64-year-old male with pulmonary emphysema **(C)** 46 days after symptom onset: subpleural reticular opacities in both lung and crescent-shaped consolidations in the left lung **(D)** after tocilizumab administration (illness day 54): enlargement and denser of bilateral pulmonary lesions. Case 3, a 47-year-old male with diabetes, chronic hepatitis and diffuse large B-cell lymphoma **(E)** 32 days after symptom onset: bilateral multiple patchy high-density opacities with bronchovascular bundle thickening **(F)** after tocilizumab administration (illness day 41): pneumonia invaded all through the right lung and left lower lobe. Case 4, a 73-year-old male with hypertension and diabetes **(G)** 21 days after symptom onset: pneumonia infiltration occurred in multi-lobes of the double sides **(H)** after tocilizumab administration (illness day 24): increased in density in the left lung and pneumothorax in the left lung.

All radiological images of the four patients were examined, and the serum specimen was obtained. A chest CT scan was taken for Case 1 after 8 days of treatment with TCZ, which indicated that the bilateral peripheral opacifications progressed to streaky consolidation with internal bronchovascular bundle thickening, while a small pleural effusion was absorbed in the right lower lung (Figure 1A–B). Case 2’s chest CT image showed bilateral subpleural reticular opacities and enlarged and denser subpleural crescent-shaped consolidations (Figure 1C & D). Six days after Case 3’s administration, pneumonia was found to have invaded the left upper lung, as shown in the x-ray image. Case 3 deteriorated rapidly with invasive mechanical ventilation on Day 8 of hospitalization and died with the development of ARDS and disseminated intravascular coagulation at the hospital on Day 20 (45 days after disease onset) (Figure 1E & F). Case 4 developed a pneumothorax under the support of mechanical ventilation and died of ARDS on Day 22 of hospitalization (Figure 1G & H).

**Figure 2.**
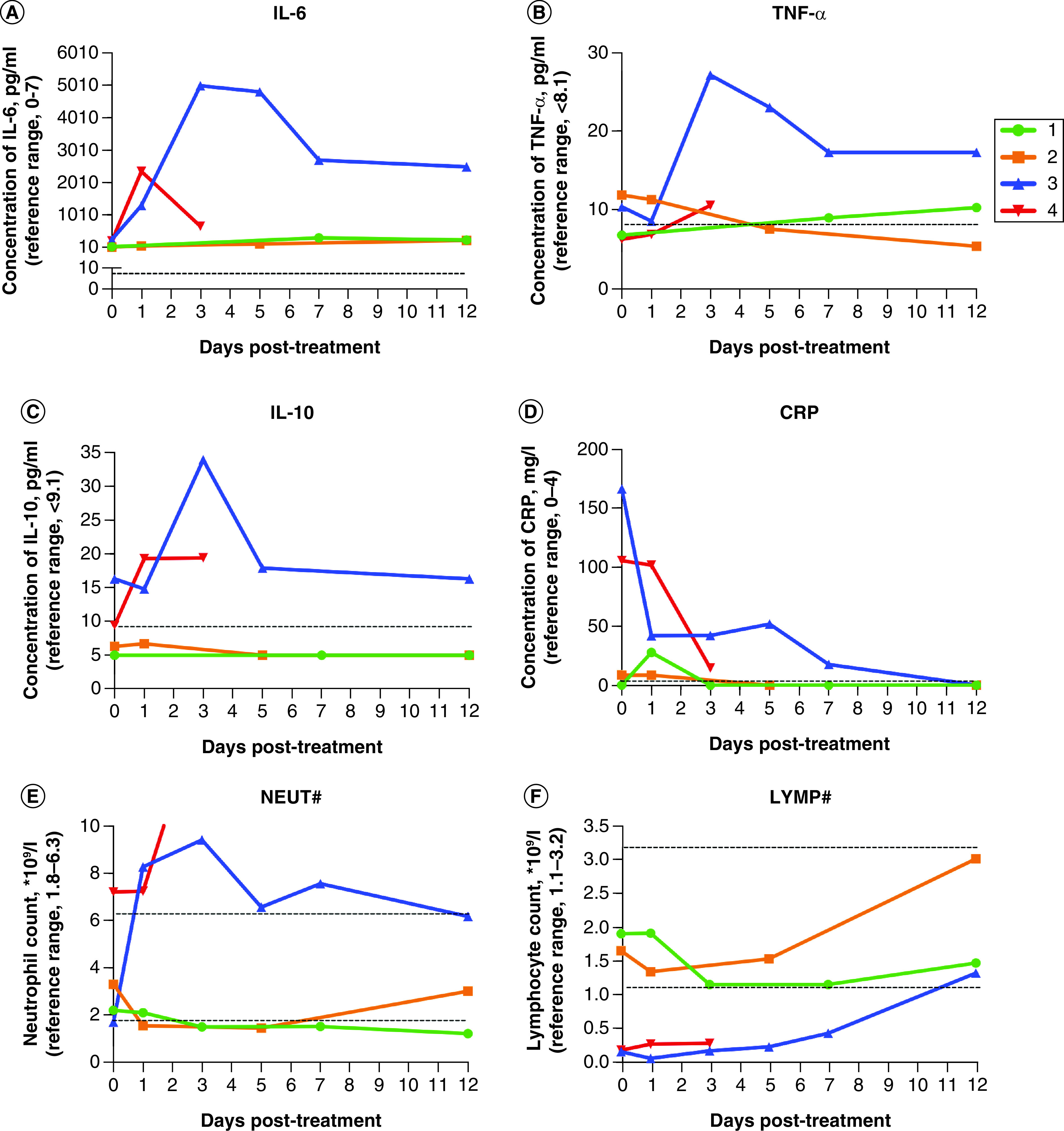
Changes of cytokines IL-6, TNF-α, IL-10 and inflammatory markers CRP and count of neutrophil and lymphocyte before and after the treatment of tocilizumab. **(A–D)** Changes of IL-6 TNF-α, IL-10 and CRP in day 0, day 1, day 3, day 5, day 7, day 12 following intravenously infusion of tocilizumab in four cases. The reference range was below the dashed line. **(E–F)** Changes of TNF-α in day 0, day 1, day 3, day 5, day 7, day 12 following intravenously infusion of tocilizumab in four cases. The reference range was between the dashed line. CRP: C-reactive protein.

The two severe patients showed elevated levels of neutrophils and reduced amounts of lymphocytes (Figure 2). The baseline level of serum IL-6 in the two moderate patients was lower than in the other two patients. Serum IL-6 indicated a surge 1–3 days after administration, followed by a decrease, though it still maintained a high level. Cytokines of TNF-α and IL-10 were shown with the same trend at a low level. CRP illustrated a downward tendency.

## Discussion

This report describes TCZ as salvage therapy that failed to respond to severe patients with COVID-19 infection, in the beginning when TCZ was first introduced in the guidelines. In our clinical findings, two severe patients died without clinical improvement. As for the other two with mild symptoms, one showed inconspicuous amelioration, and the other displayed progression of their radiological abnormalities. We observed that after exposure to TCZ, the tendency of IL-6 was rising up at the beginning and subsequently declined but maintained a relatively high level in the two severe cases. This phenomenon of temporary increase is in accordance with the ‘Bathtub theory’ mechanism that had been found previously [12]. A potential explanation is that unbound IL-6 could undergo transient accumulation at the start of the drug use due to its receptor occupation. IL-6R inhibition had no direct impact on the level of serum IL-6 in our two moderate patients. The tentative administration of TCZ in the two moderate patients was based on the long duration of their symptoms and their IL-6 levels being three-times higher, indicating that IL-6 might not be a key factor in conditions where clinical manifestations are stable in spite of slightly increased IL-6 and CT abnormalities.

TNF-α and IL-10 exhibited a similar trend and a small scope of variation compared with the normal range. The two counterparts maintained a balance between each other. Meanwhile, the inflammatory biomarker CRP illustrated a downward tendency. The CRP may indicate a sufficient dose of TCZ rather than acting as a simple disease activity index [13].

The IL-6 antagonist is recommended to treat severe patients with COVID-19 who appear to have a feature of a cytokine storm syndrome. The two severe cases in our report died after the administration of TCZ. A beneficial outcome was not achieved by the anticytokine storm treatment. Mechanical ventilation was applied to the two severe patients, which can make patients more prone to developing bacterial infections. Case 4 suffered from a pneumothorax with invasive ventilation and may have died of a co-infection of bacteria and the virus. ECMO was implemented for Case 3, who died 2 days later. The patient died from disseminated intravascular coagulation as well, with coagulation parameters such as increased D-Dimer and a marked diminishment of FIB. This phenomenon may indicate that attention should be given early on to changes in coagulation biomarkers, which might result in the rapid progress of and death from COVID-19 due to an impaired vascular endothelial cell expressing ACE2 extensively [14].

Further, we found that these two severe patients experienced increased amounts of neutrophils and fewer lymphocytes compared with the reference range, which was in line with the findings in a previous study of 201 cases [15]. In the study by Wu *et al.*, they indicated that patients with progressive neutrophils are more inclined to develop ARDS and die. Neutrophils are the primary source of chemokines and pro-informatory cytokines. The elevation of IL-6 was rising after the treatment of TCZ, which was in part due to the instant response of the agent, and was also in part due to the elevated neutrophils. Besides this, decreased counts of lymphocytes were also observed in both severe cases of our study, which could be another factor to upgrade their disease severity.

In the current study, SARS-CoV-2 was not detectable on admission although they were confirmed by PCR when they were first diagnosed, which infers that the secondary infection or inflammatory cascade outweighs the virus invasion itself. However, whether inhibition of immune response is a viable choice at this time is still debatable. IL-6 has been recognized as the stimulant of the inflammatory response in a variety of autoimmune diseases, exacerbating the condition by promoting downstream cytokines secretion. It is closely related to pulmonary inflammation and extensive lung damage and may contribute to the CRS, an assumed leading cause of death at the late stage of COVID-19 [16,17]. Wang *et al.* found that alveolar macrophages as the target cells were activated by the S protein of SARS-CoV-2, triggering the cytokine storm syndrome, which speaks in favor of the IL-6/IL-6R antagonist as a potential therapeutic approach [18]. Clinical trials have shown the efficacy of TCZ in severe COVID-19 [8–11].

However, we did not observe the effect of the TCZ on these patients nor obvious adverse events. Further, there is evidence that IL-6 is crucial for improving the survival of lung epithelial cells in mice infected with influenza virus [19,20]. Though many studies have found that increased levels of IL-6 associated with cytokine storm cascade existed in severe patients with COVID-19, the relationship between the CRS and the occurrence of ARDS is still unclear. More clinical evidence has indicated that serious infections have been found with a combination of the virus and bacteria in COVID-19 patients with long disease durations, which also can trigger the CRS and result in the deterioration of lung functions and sudden death. TCZ, as an immunosuppressive agent, may weaken the immune response to the severe infection and increase the risk of death, especially when used in the late stage of COVID-19.

The failure outcome of the treatment might be due to the patients’ long disease durations and we assumed that TCZ was administered in the midst or late stage of the patients experiencing cytokine storms, rather than in the early stage. Thus, whether TCZ exerts a positive role of immunosuppression or accelerates the disease progression is unclear. Patients were given methylprednisolone before TCZ; whether TCZ augments the function of immunosuppression without hyperinflammation was suspicious. We speculate that the optimal timing of intravenous infusion of this drug might be missed, and applying the agent at an inappropriate stage might curb the host inflammatory immune response confronting a secondary bacterial infection. Early intervention with TCZ may be more effective than application amid a cytokine storm cascade.

Hyperglycemia was another risk factor which would impair the therapeutic effect of TCZ in severe COVID-19 patients. The condition of patient 1 with diabetes was stable, while the blood glucose of patient 4 was not well-controlled. Hyperglycemia itself not only triggers an inflammatory response resulting in deteriorated COVID-19 disease, but overactivates the CRS, a negative prognostic factor for survival in severe patients [21]. Optimal control of glycemia in this kind of case subset needed to be considered. In addition to diabetic history, patient 4 was also suffered from long-term hypertension. The use of ACE inhibitors or angiotensin receptor blockers may interfere with ACE2 expression and its activity, which is a high risk factor and may make patients prone to be infected by SARS-CoV-2. Disruption of ACE2 pathways leads to worse prognosis by endothelial dysfunction, prothrombotic status and disseminated coagulopathy which could confer a worse prognosis [22,23].

Serious adverse events including osteonecrosis of the jaws, severe infections, gastrointestinal reactions, etc. may worsen the condition of the patients [24]. In our cases, no safety issues were identified. More large-scale randomized clinical trials are required to evaluate the efficacy and safety of IL-6 antagonist in treating patients with COVID-19.

The study has several limitations. First, this was a small case series with quite heterogeneous patients, and all patients were transferred to the designated hospital after a long period of symptom onset. Thus, first-hand information was not obtained. Second, the administration of TCZ in two moderate patients took place within a tentative treatment. It might not seem suitable for such cases to benefit. Third, all patients were treated with multiple other medications, and the role of the interactions of these agents was not elucidated. At last, the optimal timing within the whole course of the disease required to achieve clinical benefits from the TCZ still needs to be clearly understood.

Given the limited benefit our patients received, we suggest that patients’ clinical conditions, comorbidities and disease severity should be taken into consideration to improve clinical decision making. Early intervention with TCZ may be more effective than application amid a cytokine storm cascade. More large-scale randomized clinical trials are required to evaluate the efficacy and safety of IL-6 antagonist in treating patients with COVID-19.

**Summary points**

- Coronavirus disease 2019 (COVID-19) is a life-threatening disease and how to decrease the high mortality is of great importance.
- Four patients were administrated with tocilizumab, an IL-6 receptor antagonist to determine whether it shows favorable results in two severe COVID-19 patients as well as in two moderate patients with high levels of serum IL-6 who attained no benefit from standard care.
- There was no much difference in the clinical feature improvements and computed tomography images in two moderate patients after treatment of IL-6R antagonist. The other two severe patients presented with recurrent fever and needed ventilation in intensive care unit and died of disseminated intravascular coagulation and acute respiratory distress syndrome, respectively.
- Administration of tocilizumab was not shown to have a favorable outcome in this preliminary uncontrolled case series. Early identification of cytokine release syndrome in the treatment of COVID-19 patients with IL-6 antagonist is required.
